# A first look into radiomics application in testicular imaging: A systematic review

**DOI:** 10.3389/fradi.2023.1141499

**Published:** 2023-04-17

**Authors:** Salvatore C. Fanni, Maria Febi, Leonardo Colligiani, Federica Volpi, Ilaria Ambrosini, Lorenzo Tumminello, Gayane Aghakhanyan, Giacomo Aringhieri, Dania Cioni, Emanuele Neri

**Affiliations:** Department of Translational Research, Academic Radiology, University of Pisa, Pisa, Italy

**Keywords:** testicular imaging, radiomics, radiomics quality score, germ cell tumors, seminoma, nonseminoma, gonadal function

## Abstract

The aim of this systematic review was to evaluate the state of the art of radiomics in testicular imaging by assessing the quality of radiomic workflow using the Radiomics Quality Score (RQS) and the Quality Assessment of Diagnostic Accuracy Studies-2 (QUADAS-2). A systematic literature search was performed to find potentially relevant articles on the applications of radiomics in testicular imaging, and 6 final articles were extracted. The mean RQS was 11,33 ± 3,88 resulting in a percentage of 31,48% ± 10,78%. Regarding QUADAS-2 criteria, no relevant biases were found in the included papers in the patient selection, index test, reference standard criteria and flow-and-timing domain. In conclusion, despite the publication of promising studies, radiomic research on testicular imaging is in its very beginning and still hindered by methodological limitations, and the potential applications of radiomics for this field are still largely unexplored.

## 1. Introduction

Radiomics is defined as the process of obtaining high-dimensional data from medical images as quantitative features (1). In recent years, a growing interest in this field led to a rising number of applications in many different medical imaging fields (2). Radiomics has gained importance especially in the field of precision medicine, which aims to tailor treatments based on specific characteristics, including genetical and phenotypical ones (3). The huge number of quantitative features obtained through Radiomics may be selected and used for classification, prediction and prognosis of different neoplasms, providing additional information about tumor phenotype and gene expression pattern (4, 5). However, application of radiomics is still mainly limited to research setting for many reasons, mostly due to the lack of reproducibility and repeatability of the results, often associated with heterogeneities in the several steps of radiomics workflow (6). Thus, effective evaluation criteria and standardization of radiomic workflows are needed, ranging from the data collection to the model building (7). An attempt to standardize research in radiomics was made by Lambin et al., who developed the Radiomics Quality Score (RQS) for quality assessment of radiomics studies (8). Undoubtedly, oncologic imaging is the main field for radiomics application, including testicles (9–13).

Testicular cancer is a relative rare disease (representing only the 1% of neoplasm and the 5% of urological tumors in males) and it is predominant in young/middle-aged males (14, 15). Many imaging techniques can be used for the evaluation of testicles in different clinical scenarios, before and after therapy (surgery, chemotherapy or radiotherapy), and include ultrasound (US), magnetic resonance imaging (MRI), computed tomography (CT) and fluorodeoxyglucose-positron emission tomography (FDG-PET) (16). The characterization of histologic type is of extreme importance as different tumors (e.g., seminoma germ cell tumor and non-seminoma germ cell tumor) present different prognosis and treatment. Moreover, orchidectomy is still the standard of care for testicular cancer, although it may have a negative impact on reproduction (17). In this setting, alternative non-invasive methods of diagnosis had been proposed to avoid unnecessary surgery, including MRI for the identification and differentiation of benign scrotal lesions, but it still may be inconclusive (18). Additionally, imaging plays an important role in the diagnostic framework of male infertilities, as a relationship between US testicular volume and testicular steroidogenic function has already been demonstrated (19, 20). However, the lack of standardized method to calculate US testicular volume and validated reference ranges has prompted the search for other reliable quantitative US parameters (21). The aim of this systematic review is to evaluate the state of the art of radiomics in testicular imaging by assessing the quality of radiomic workflow.

## 2. Materials and methods

### 2.1. Literature search

To identify records of interest, two reviewers (S.C.F. and M.F.) independently performed a systematic literature search for potentially relevant articles about Radiomics applications in testicular imaging.

The examined medical literature archives were PubMed, Scopus, and Web of Science, using the following search terms: testicular AND radiomics. Filters were applied to include only original research published in English. No restrictions in country of publication, study design, and outcomes were applied. The last search was run in September 2022. Duplicates have been removed and all the selected articles were initially screened reviewing the titles and abstracts. After the screening, the authors read the full text of the studies and any disagreement was overcome by discussion to reach a mutual agreement. From each study, the following data were extracted: publication year, number of patients, study design, study aim, journal topic, and professional role of the first author.

### 2.2. Study evaluation

The methodological quality of the included studies was carried out by two readers (M.F. and L.C.) using the Radiomics Quality Score (RQS), as proposed by Lambin et al., and the Quality Assessment of Diagnostic Accuracy Studies-2 (QUADAS-2), as proposed by Whiting et al. (8, 22). Conflicts between the two reviewers were resolved in consensus together with a third reviewer (S.C.F).

Radiomics Quality Score (RQS) is a tool made up of 16 items, categorized by Park et al. into 6 domains, with different possible scores in relation to its importance (23). According to the RQS, the summed total score ranges from −8 to 36. To calculate percentages, a score of 0% was assign to studies with summed score from −8 up to 0, while a score of 100% was defined for summed score of 36. QUADAS-2 criteria were used to assess presence of relevant biases, including the following domains: patient selection, index test, reference standard and flow and timing. For each domain, the risk of biases and concerns regarding the applicability of the review question were scored as low, high or unclear (in case of insufficient data). You may insert up to 5 heading levels into your manuscript as can be seen in “Styles” tab of this template. These formatting styles are meant as a guide, as long as the heading levels are clear, Frontiers style will be applied during typesetting.

## 3. Results

**Figure 1:**
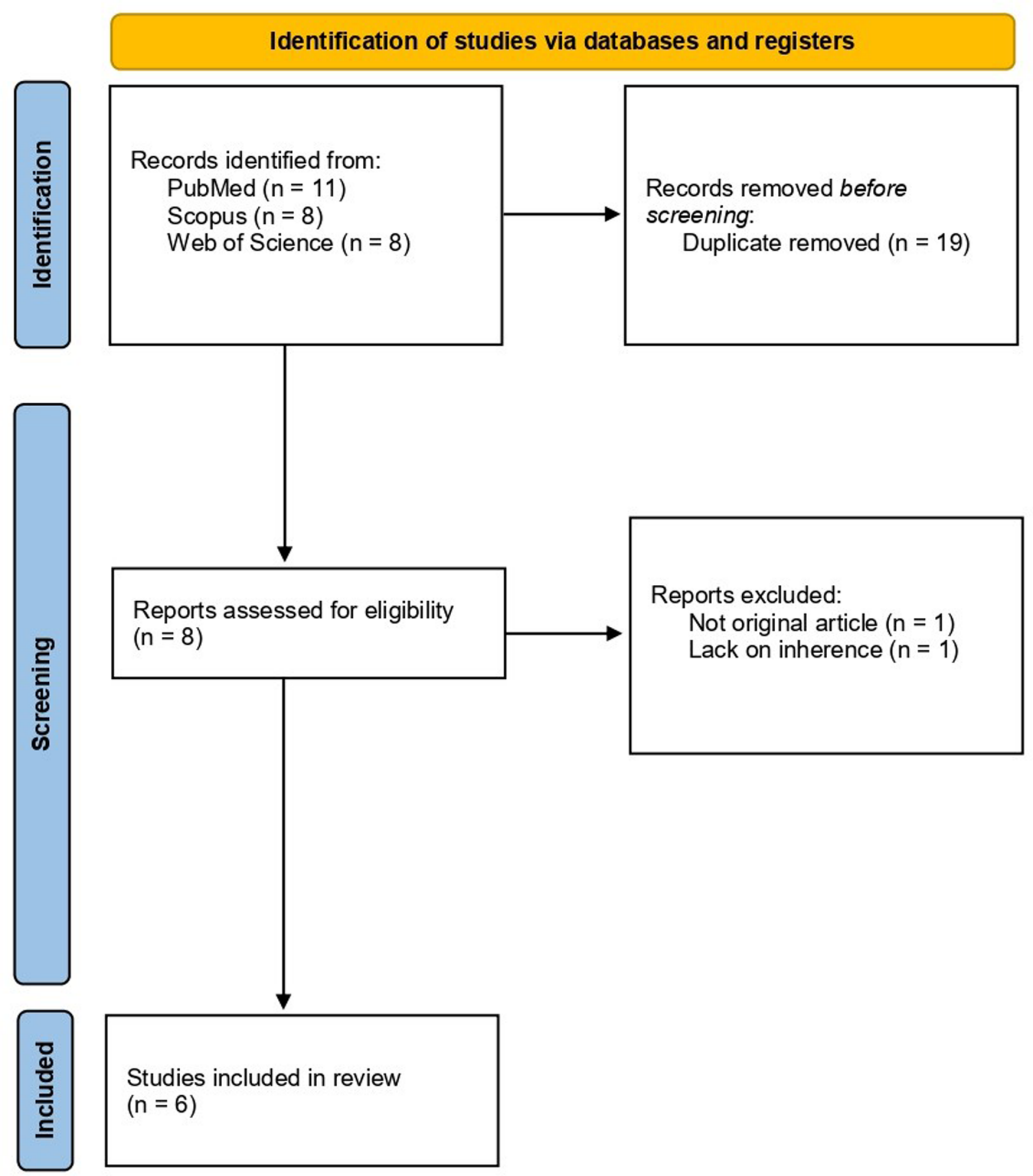
Study selection process flow-diagram according to PRISMA statement 2020 (24).

After the exclusion of duplicates (19) and unrelated papers (2), 6 articles were finally included in the review. The study selection flow-chart is resumed in Figure 1.

Out of 6 articles, none were published before 2018, four (66%; 4/6) were published in clinical journals and only two (33%; 2/6) in radiological journals. The mean patient number was 70 ± 23,86 (range 39–97). Many of the studies had a retrospective study design (83%; 5/6), while only one was a prospective observational study (17%; 1/6). In half of the papers included in the review, the first author was a radiologist. Most of the articles addressed the oncologic topic (83%; 5/6), focusing on differential diagnosis between benign and malignant testicular masses or prediction of lymph nodes histopathology after chemotherapy. Only one article investigated potential role of radiomics as gonadal function biomarker. Characteristics of included articles are resumed in Table 1.

**Table 1:** Characteristics of included articles.

First Author	Publication year	Patients	Study design	Study aim	Imaging modality	Journal topic	Professional role of the first author	Segmentation strategy	ML	Features type
Baessler (25)	2020	80	Retrospective	Histopathology prediction of lymph nodes after post-chemotherapy LN dissection in metastatic non-seminomatous testicular germ cell tumors.	CT	Radiological	Radiologist	Semi-automatic	Yes	First order and higher order
De Santi (26)	2022	85	Prospective observational	Extraction of radiomics US features correlating with testicular function.	US	Clinical	Not radiologist	Manual	Yes	First order and higher order
Fan (27)	2022	97	Retrospective	Development of a radiomics signature of ADC for discriminating between benign and malignant testicular masses and compare its performance with minimum and mean ADC.	MR	Radiological	Radiologist	Manual	Yes	First order and higher order
Feliciani (28)	2021	42	Retrospective	Pre-operative prediction of testicular neoplasm histology through an MRI-based Radiomics signature.	MR	Clinical	Not radiologist	NR	Yes	First order and higher order
Lewin (29)	2018	77	Retrospective	Pre-operative identification of fibrosis in lymph nodes post-chemotherapy.	CT	Clinical	Not radiologist	Manual	Yes	First order and higher order
Zhang (30)	2019	39	Retrospective	Differentiation between seminomas and non-seminomas through a T2-weighted image (T2WI)-based radiomics signature.	MR	Clinical	Radiologist	Manual	No	First order and higher order

ML, machine learning; NR, not reported.

Overall, the included articles achieved a mean RQS total of 11,33 ± 3,88 (range 6–18) and a percentage of 31,48% ± 10,78% (range 16,67%–50%). Imaging protocols, features reduction, discrimination statistics and comparison to gold standard are well-documented in all the included articles. Validation without retraining was performed in all the studies, even though only on dataset from the same institute. Half of the studies reported multiple segmentation, to analyze feature robustness to segmentation variabilities, while only two provided more holistic models combining radiomics with clinical variables. None of the studies performed phantom studies or imaging at multiple time-points. Similarly, no study reported cut-off analyses, calibration statistics, decision curve or cost-effectiveness analysis. Finally, none of the included articles made code and data publicly available to facilitate reproducibility of the study. The detailed RQS score for all included articles for each RQS item is shown in Table 2.

**Table 2:** Radiomics quality score (RQS) for included papers.

First Author (year)	Item 1	Item 2	Item 3	Item 4	Item 5	Item 6	Item 7	Item 8	Item 9	Item 10	Item 11	Item 12	Item 13	Item 14	Item 15	Item 16	RQS (total)	RQS (%)
Baessler (2019)	1	1	0	0	3	0	1	0	2	0	0	2	2	0	0	0	12	33,33
De Santi (2021)	1	0	0	0	3	1	1	0	1	0	7	2	2	0	0	0	18	50,00
Fan (2022)	1	1	0	0	3	0	0	0	2	0	0	2	2	0	0	0	11	30,56
Feliciani (2021)	1	0	0	0	3	0	1	0	2	0	0	2	2	0	0	0	11	30,56
Lewin (2018)	1	0	0	0	3	1	1	0	2	0	0	2	2	0	0	0	12	33,33
Zhang (2019)	1	1	0	0	3	0	0	0	1	0	0	2	2	0	0	0	10	27,78

**Figure 2:**
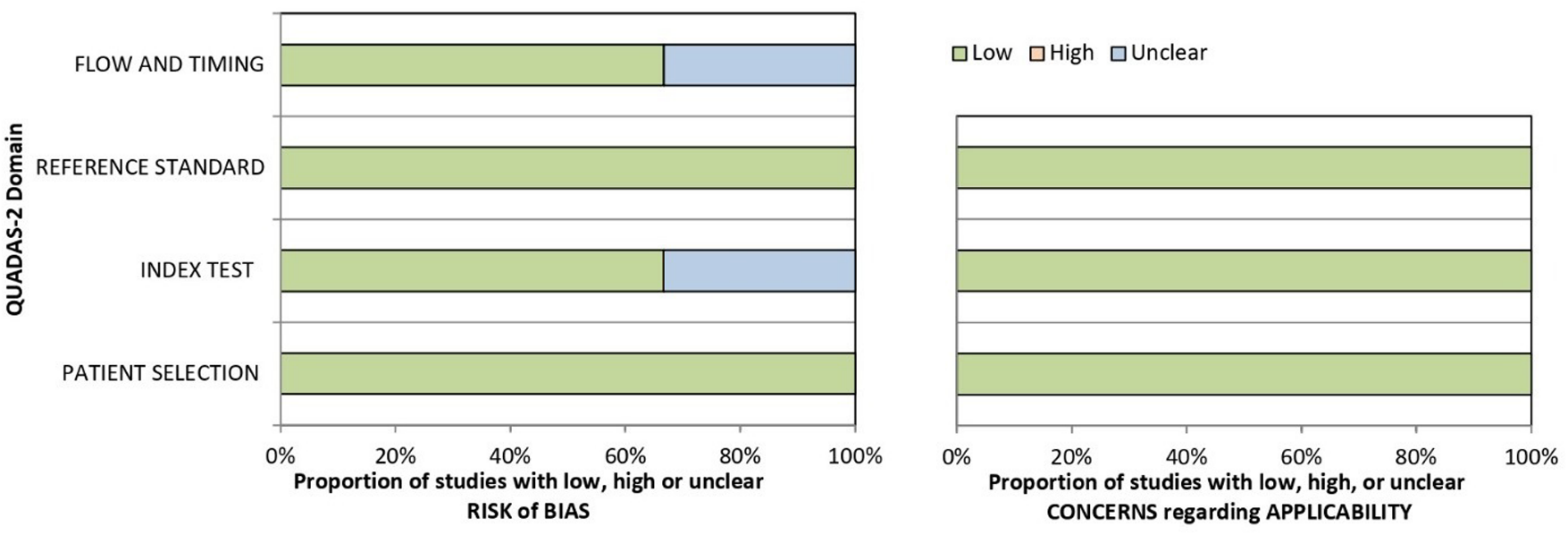
Risk of bias and concerns regarding applicability histograms according to QUADAS-2 for included papers.

The risk of bias and applicability concerns according to the QUADAS-2 are summarized in Table 3 and Figure 2. All studies were rated as low risk regarding patients' selection and reference standard interpretation. Regarding test interpretation, 4 studies (66%; 4/6) were rated as low, and two studies as unclear (unclear data). Finally, regarding flow and timing of the study, 2 studies (33%; 2/6) were considered unclear.

**Table 3:** QUADAS-2 for included papers.

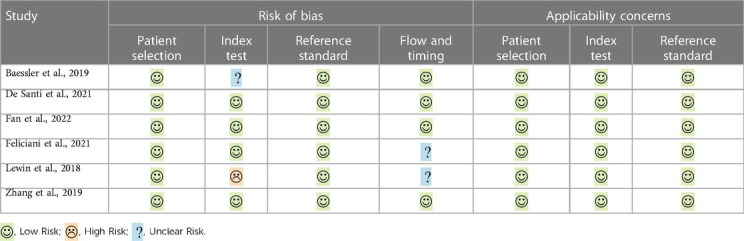

## 4. Discussion

Radiomics is a new engineering approach based on automated high-throughput extraction of quantitative features from medical images (2). Radiomics-based models may empower radiology to overcome the limit of radiologists' visual interpretation.

Despite promising studies, the potential applications of radiomics for testicular imaging are still largely unexplored. Indeed, testicular tumors are relatively rare, as they represent only the 1% of neoplasm and the 5% of urological tumors in males (14, 15). Coherently, the number of patients enrolled in the included studies was very low (range 39–97). However, it is conceivable that the interest in radiomics application will increase in the following years considering the estimated rise by 24% of testicular cancer incidence in the years from 2005 to 2025 (31). In the preoperative setting, it will become more and more important to precisely identify patients with testicular benignities, accounting for approximately the 20% of testicular masses, to avoid inappropriate radical inguinal orchiectomy (32, 33). Aimed at accurately discriminating between benign and malignant masses, Fan et al. developed an ADC-based radiomics signature and compared its classification performance with that of minimum and mean ADC (27). No statistically significant difference was found for this parameter between benign and malignant testicular masses (34), probably because the mean ADC does not take into account the whole lesion heterogeneity. Conversely, ADC-based radiomics signature provided an optimal performance in validation cohort (AUC 0.868). However, to solely discriminate between benign and malignant masses may not be enough in patients unwilling to undergo orchiectomy. In this cohort of patients, the therapeutic strategy usually relies on histological subtype.

The most common testicular cancers are testicular germ tumors (TGCTs), accounting for approximately the 90%–95%, and are split into two categories: seminomas (SGTs) and non-seminomas (NSGTs) (35). The main difference between SGTs and NSGTs lies in the different sensitivity to radio- and chemo-therapy. Invasive procedures, such as biopsy, are not currently recommended in order to avoid tumor spread (33). Thus, imaging may have a role to differentiate the two histological subtypes and decide the optimal therapeutic strategy (36). To exploit all the potential value of MRI in this clinical setting, Zhang et al. applied radiomics to T2-weighted (T2W) sequence to differentiate SGTs and NSGTs. A radiomics signature with five different features achieved an AUC of 0.979 (30). Feliciani et al. extended the work of Zhang by investigating the diagnostic performance of MRI and radiomics model in differentiating between TGCTs and testicular non germ cells tumors (TNGCTs). T2W-based radiomics model achieved an overall accuracy of 89% in differentiating these two categories. Also, an optimal performance in discriminating between SGCTs and NSGCTs was confirmed (28).

Another relevant difference between SGCTs and NGCTs is that nearly half of NSGCTs already show metastases at the time of first diagnosis (37). Currently, the standard of care for these patients is chemotherapy followed by post-chemotherapy retroperitoneal lymph node dissection (pcRPLND) of residual nodal masses with measurements > 1 cm and oncologic markers plateau or normalization (38). However, after pcRPLND, viable cancer is detected in only 15% and teratoma in 40% of patients, while the remaining show fibrotic or necrotic tissues (39). Radiomics may provide imaging biomarkers indicating which patient would actually benefit from pcRPLND in order to reduce overtreatment of young patients.

Baessler et al. trained a machine learning classifier to differentiate “benign” (fibrotic/necrotic) from “malignant” (viable cancer/teratoma) lymph nodes on contrast-enhanced CT in patients with NSGCT post-chemotherapy. The classifier achieved an accuracy of 0.81 and outperformed the commonly used “size” criterion (0.68) (25). The model performance in discriminating fibrotic or necrotic lymhp nodes from neoplastic ones may be further improved through the combination of radiomics features with already established clinical biomarkers. Indeed, Lewin et al. optimized a radiomics-based classifier performance by adding clinical variables to the model, such as pre-chemotherapy biomarkers, and achieved the best algorithm performance (AUC 0.88) (29).

Beyond oncology, radiomics have also demonstrated the potential role in providing valuable and reliable imaging biomarkers of gonadal function. Specifically, radiomics may empower US as an *in vivo* imaging measurement of testicular function. The unavailability of standard methods and reference ranges for US testicular volume measurement prompts to look for other parameters, such as testicular echostructure (40–42) However, despite promising results, the clinical applicability of testis echostructure was limited by the operator dependency of its measurement and by the lack of a widely accepted quantitative measure (43).

De Santi et al. designed a prospective observational study to correlate objective US features with both spermato- and steroido-genesis. First, the authors demonstrated that US texture features significantly predict visually defined inhomogeneity, providing for the first time a reliable mathematical quantification of a subjective US evaluation. Second, thirteen US texture features significantly predict sperm concentration, total sperm number, progressive motility, total motility and sperm morphology, while no significant correlation was found with total testosterone serum levels. Finally, at classification analysis, US textural features were validated as parameters able to classify patients' accordingly to semen parameters alterations (26). However, despite the growing interest in radiomics, the methodology's complexity and the uncertain quality of these studies are significantly slowing down the implementation of these techniques in the clinical practice (44, 45).

The methodological quality of the 6 included studies was assessed using RQS and QUADAS-2. The mean RQS was 11,33 ± 3,88 (range 6–18) resulting in a percentage of 31,48% ± 10,78% (range 16,67%–50%). This percentage is slightly higher compared to the average result (18.87%) reported by Spadarella et al. in a systematic review of RQS applications. However, it would be premature to draw firm conclusions from this difference, as it could be largely explained by the limitations of the RQS tool itself and particularly by its lack of reproducibility (46–48). Regarding the items of RQS, it's worth pointing out many key points particularly given the fact that radiomics application in testicular imaging is in its very beginning. First, none of the studies have performed an external validation without retraining. The lack of external validations significantly questions the credibility of the model performance if transposed to real clinical practice (8). As the number of studies regarding this topic increases, it can be expected that more and more institutes will be interested in collaborating leading to increased number of external validations. Unfortunately, none of the studies have provide public code or data to facilitate the reproducibility of the studies and, most importantly, starting to promote further research in this clinical setting. Finally, one study out of six presented a prospective observational design. Prospective studies provide the highest level of evidence regarding the real clinical value of radiomics also providing essential information about its cost-effectiveness ratio.

Regarding QUADAS-2 criteria, no relevant biases were found in the included papers in the patient selection, reference standard criteria and flow-and-timing domains.

In conclusion, radiomics application in testicular imaging is a promising area of research, despite being in its early stages, and should be pursued to improve the accuracy of testicular tumor diagnosis, staging, and treatment planning. Compared to other oncologic diseases, invasive procedures such as testicular biopsy are not recommended, and currently represent a limitation in the pre-operative characterization of testicular cancer and therapy planning. Radiomics has already been shown able to fill this gap when applied to cross-sectional imaging, but it could be even more useful if employed in ultrasound, which represents a first-line diagnostic imaging technique. However, clinical implementation is hindered by methodological limitations, including the lack of external validation and the prevalence of retrospective studies. Large-scale multicenter studies and prospective designs are needed to overcome these limitations and push this field forward.
